# Non-targeted metabolomic analysis of non-volatile metabolites in a novel Chinese industrially fermented low-salt kohlrabi

**DOI:** 10.3389/fnut.2024.1450789

**Published:** 2024-08-30

**Authors:** Xiaohan Jia, Xinyi Wang, Hongfan Chen, Dayu Liu, Bo Deng, Ling Ao, Jianping Yang, Xin Nie, Zhiping Zhao

**Affiliations:** ^1^College of Food and Biological Engineering, Chengdu University, Chengdu, China; ^2^College of Culinary and Food Science Engineering, Sichuan Tourism University, Chengdu, China; ^3^Solid-State Brewing Technology Innovation Center of Sichuan, Luzhou, China

**Keywords:** kohlrabi, low-temperature and low-salt fermentation, LC-MS/MS, differential metabolites, metabolic pathways

## Abstract

Low-temperature and low-salt fermented Chinese kohlrabi (LSCK) represents a novel approach to producing low-salt kohlrabi without the need for desalination during processing, as compared to traditional techniques. However, the profile of its non-volatile metabolites remains unclear. In order to investigate the non-volatile metabolites and their changes in LSCK during fermentation, the LSCKs fermented for 0 day (0D), 45 days (45D) and 90 days (90D) were analyzed using LC-MS/MS non-targeted metabolomics coupled with multivariate statistical analysis. The results showed that 60, 74, and 68 differential metabolites were identified in the three groups A1 (0D and 45D), A2 (0D and 90D), and A3 (45D and 90D) (VIP >1, *p* < 0.05, Log2FC >1), respectively. The differential metabolites were mainly amino acids, peptides, and analogues, fatty acyls, organic acids and derivatives, and carbohydrates and carbohydrate conjugates. Seventeen common differential metabolites were identified in A1, A2, and A3 groups. Kyoto Encyclopedia of Genes and Genomes (KEGG) analysis suggested that the alanine, aspartate and glutamate metabolism, butanoate metabolism, α-linolenic acid metabolism, arginine biosynthesis, and phenylalanine metabolism were significantly correlated with the differential metabolites. The present study elucidates for the first time the changes in non-volatile differential metabolites and their associated metabolic pathways in the novel Chinese low-salt kohlrabi, providing a theoretical basis for improving the industrial fermentation process of this innovative product.

## 1 Introduction

Kohlrabi (*Brassica juncea* var. *megarrhiza* Tsen et Lee), a member of the cruciferous *Brassica* annual herb family, is widely cultivated in China. Known for its high nutritional value, kohlrabi is rich in vitamins, proteins, and carbohydrates. It also serves as a quality source of biologically active components, such as thioglucosides and indole derivative (1). However, raw kohlrabi has high levels of isothiocyanate, which gives a strong mustardy and bitter flavor and makes it unsuitable for direct consumption. After fermentation, kohlrabi develops a moderately sweet and salty taste with a robust soy sauce flavor (2). Fermented kohlrabi is a renowned Chinese specialty and is one of the four most famous pickles in Sichuan Province, alongside mustard tubers, Dongcai, and mustard vein (3).

Traditional fermentation methods for kohlrabi often use a high salt concentration (15–20%, w/w). Long-term consumption of high-salt foods can elevate sodium ion levels in the human body, resulting in sodium-potassium imbalance and thus leading to diseases such as hypertension and atherosclerosis (4). Consequently, desalination is required to produce low-salt kohlrabi. However, this process not only causes a significant loss of nutrients such as proteins but also increases production costs for treating sodium-containing wastewater. Therefore, low-salt fermentation techniques are gaining global attention. Salt plays a crucial role in forming food taste and flavor (5). Liang et al. (6) reported that a 6% salt concentration in Chinese sauerkraut fermentation promoted higher lactic acid bacteria abundance and better texture. Fermentation temperature also affects the physicochemical properties of fermented products by influencing microbial metabolites. For instance, Aung and Eun (7) found that laver fermented at 25°C produced more flavonoids and enhanced α-amylase inhibitory activity than that fermented at 30°C.

Compared to traditional kohlrabi, the production of LSCK uses less salt and does not require desalination. LSCK has higher protein and reduced sugar levels and possesses elevated concentrations of flavor substances such as alcohols, ketones, pyrazines, ethers, and nitriles, as documented in our previous study (8). Moreover, the volatile differential metabolites of LSCK were analyzed in our previous study (9). Given kohlrabi’s richness in nutritional and functional metabolites, a thorough investigation into both volatile and non-volatile metabolites of LSCK is necessary. However, there is a paucity of literature on the non-volatile metabolites and their changes in this novel low-salt, industrially fermented kohlrabi. Furthermore, the mechanisms underlying the formation of these differential non-volatile metabolites remain unclear. In this study, the non-volatile metabolites of LSCKs with different fermentation periods (0, 45, and 90 days) were analyzed using non-targeted LC-MS/MS metabolomics, and differential metabolites were screened by multivariate statistical analyses. The present study provides a theoretical basis for improving LSCK processing and utilizing its nutritional and functional metabolites.

## 2 Materials and methods

### 2.1 Preparation of the novel low-salt industrially fermented kohlrabi LSCK

The industrially fermented LSCK was prepared as described in our previous studies (9). The kohlrabies were naturally air-dried outdoors with an average temperature of 7–12°C for 20–40 days after harvesting. After air-drying, the kohlrabies were washed in water at 60 ± 2°C, then dried at 35–40°C for 15 min in a drier. The pretreated kohlrabies were then mixed with 2.5% (w/w) salt and pickled at 4 ± 1°C for 2 days. Then, 1.5% (w/w) salt was added to the once pickled kohlrabies and followed by fermentation at 4 ± 1°C for 0, 45, and 90 days, termed 0D, 45D, and 90D, respectively.

### 2.2 Metabolite extraction from LSCK

Metabolite extraction was performed as described in our previous study (10). Fifty micrograms of LSCK were transferred into a clean 2 mL-microtube with a 6 mm grinding bead. Then, 400 μL of extraction solution [methanol:water = 4:1, (v:v)] combined with 0.02 mg/mL of internal standard (L-2-chlorophenylalanine) was used for LSCK metabolite extraction. LSCK was completely ground by using the frozen tissue grinder (Wonbio-96C, Shanghai Wanbo Biotechnology, Shanghai, China) for 6 min at −10°C and 50 Hz. Subsequently, the LSCK metabolite was extracted by low-temperature ultrasonic extraction (KW-100TDV, Kunshan Shumei, Kunshan, China) for 30 min at 5°C and 40 kHz. The extracted LSCK samples were stored at −20°C for 30 min and centrifuged (H1850R, Cence, Changsha, China) at 13,000 g for 15 min at 4°C. The supernatant was used for LC-MS/MS analysis.

### 2.3 Mass spectrometry conditions

The LC-MS/MS analysis for LSCK extract was performed on a Thermo UHPLC-Q Exactive HF-X system equipped with an ACQUITY HSS T3 column (100 mm × 2.1 mm i.d., 1.8 μm; Waters, United States) at Majorbio Bio-Pharm Technology Co. Ltd. (Shanghai, China). The mobile phases were comprised of solvent A and solvent B. Solvent A was 0.1% formic acid in water: acetonitrile solution (95:5, v/v), while solvent B was 0.1% formic acid in acetonitrile: isopropanol: water solution (47.5:47.5:5, v/v/v). The separation and MS conditions were detailly described in our previous study (10). The optimal conditions were source temperature 425°C; sheath gas flow rate 50 arb and aux gas flow rate 13 arb; ion-spray voltage floating (ISVF) −3,500 V in negative mode and 3,500 V in positive mode, respectively. Normalized collision energy, 20–40–60 V rolling for MS/MS. Full MS resolution was 60,000, and MS/MS resolution was 7,500. Data acquisition was performed with the Data Dependent Acquisition (DDA) mode. The detection was carried out over a mass range of 70–1,050 m/z.

### 2.4 Statistical analysis

The LC/MS raw data was pretreated by Progenesis QI (Waters Corporation, Milford, United States) software. The non-volatile metabolites were identified by searching the primary databases HMDB,^1^ Metlin,^2^ and Majorbio Database. The R package “ropls” (Version 1.6.2) was employed to perform principal component analysis (PCA) and orthogonal partial least squares-discriminant analysis (OPLS-DA), and 7-cycle interactive validation evaluating the stability of the model. The non-volatile metabolites with VIP >1, *p* < 0.05 were considered as significantly differential non-volatile metabolites based on the Variable Importance in the Projection (VIP) obtained by the OPLS-DA model and the *p*-value obtained by Student’s *t*-test. Differential non-volatile metabolites among the A1, A2, and A3 groups were mapped into their biochemical pathways through metabolic enrichment and pathway analysis based on KEGG database.^3^ Python packages “scipy.stats”^4^ was used to perform enrichment analysis to obtain the most relevant biological pathways for experimental treatments.

## 3 Results and discussion

### 3.1 PCA analysis for LSCK non-volatile metabolites

**Figure 1:**
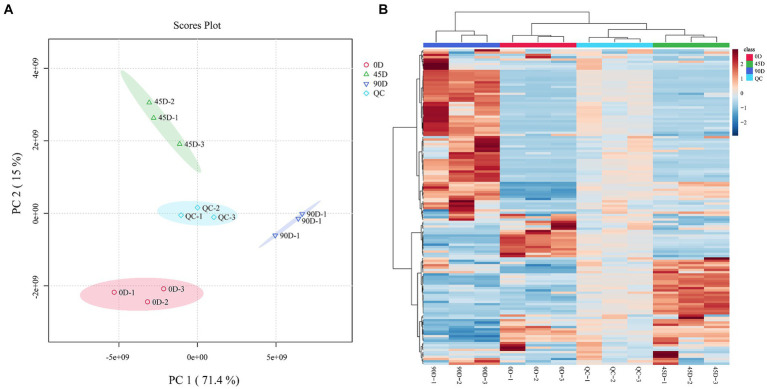
Principal component analysis (PCA) plot of the metabolites for 0D, 45D, 90D, and QC samples **(A)**, hierarchical clustering analysis (HCA) of nontargeted metabolomics for 0D, 45D, 90D, and QC samples **(B)**. 0D, 45D, and 90D represent the LSCK fermented for 0 days, 45 days, and 90 days, respectively, QC stands for quality control.

In order to reveal the effect of the fermentation period on LSCK non-volatile metabolites, the metabolite profiles of the three different kohlrabies were evaluated by LC-MS/MS coupled with multivariate statistical methods. As shown in Figure 1A, the quality control (QC) group closely distributed and clustered in the center, indicating high reproducibility and reliability of the data. The 0D, 45D, and 90D samples were distributed in distinct regions, suggesting significant differences in metabolites among the different LSCKs. Based on the PCA results, the three treatment groups were established A1 (0D and 45D), A2 (0D and 90D), and A3 (45D and 90D). PC1 contributed 71.4%, while PC2 contributed 15%, for a total contribution of 86.4%, indicating that the PCA model had good interpretability. In order to better visualize the differences between the LSCK samples, hierarchical clustering analysis (HCA) was performed in the form of heat maps, as shown in Figure 1B. It was obvious that the non-volatile metabolite profiles were significantly different among LSCKs.

### 3.2 OPLS-DA analysis for LSCK non-volatile metabolites

**Figure 2:**
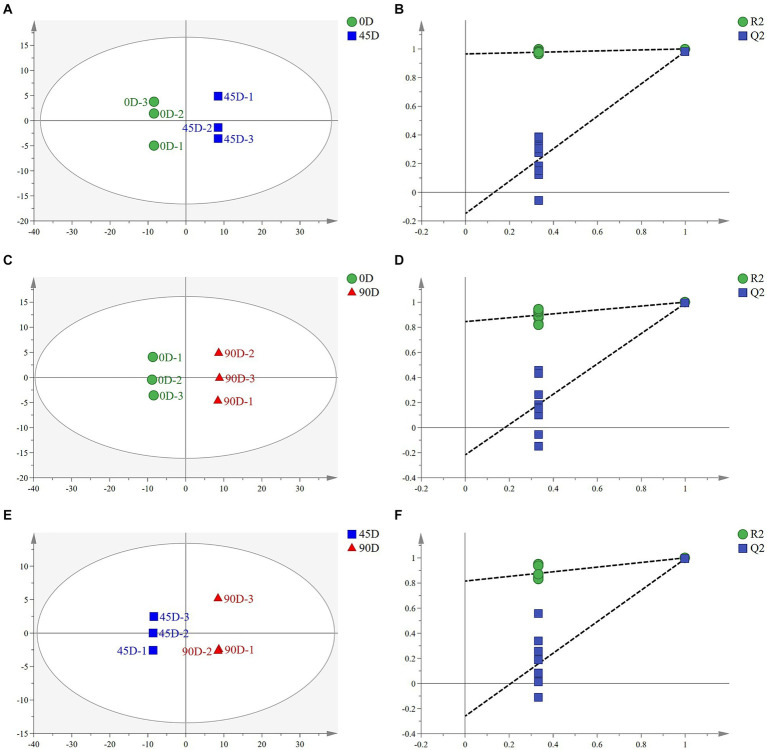
Scatter plots and permutation test of OPLS-DA models for LSCK, **(A,C,E)** indicates the OPLS-DA analysis for A1, A2, and A3, while **(B,D,F)** represents permutation test for A1, A2, and A3.

OPLS-DA analysis can better remove confounding factors unrelated to categorical information and further enhance the model’s analytical ability. Figures 2A,C,E show the plots of OPLS-DA scores for the three treatment groups, A1 (0D and 45D), A2 (0D and 90D), and A3 (45D and 90D), respectively. All three treatment groups were significantly separated from each other, suggesting that different fermentation periods caused the significant differences in non-volatile metabolite compositions and contents among the LSCKs. The parameters of the OPLS-DA model are listed in Table 1. The *R*^2^*X* scores in the model were all larger than 0.5. Moreover, the scores of *R*^2^*Y* and *Q*^2^ were both larger than 0.9, indicating the model could effectively explain and predict the differences between the metabolites in each group.

**Table 1:** Parameters of the OPLS-DA models of LSCK.

Group	*R* ^2^ *X*	*R* ^2^ *Y*	*Q* ^2^
A1 (0D–45D)	0.729	1.000	0.983
A2 (0D–90D)	0.763	1.000	0.991
A3 (45D–90D)	0.714	1.000	0.993

To further demonstrate the validity of the results, 200-loop iteration permutation tests were performed, as shown in Figures 2B,D,F. *Q*^2^ represents the predictive ability of the OPLS-DA model. All *Q*^2^ points located on the left side of the plot were lower than the original *Q*^2^ points on the right side. Furthermore, the regression lines of *Q*^2^ were all intersected with the negative half-axis of the *Y*-axis, indicating that the model had good reliability and stability. On the other hand, no over-fitting was observed.

### 3.3 Classification of non-volatile metabolites in LSCKs

**Figure 3:**
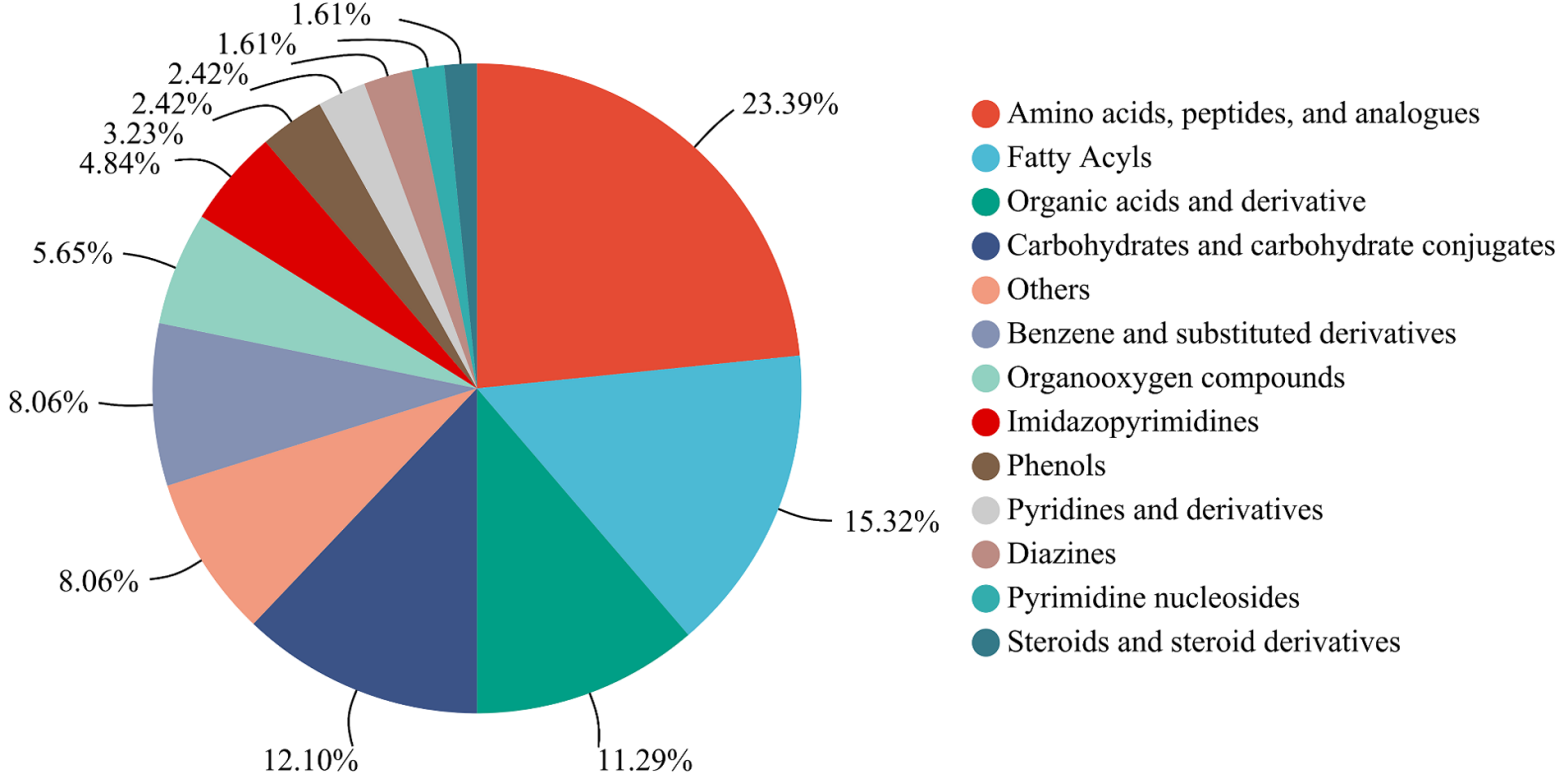
Pie chart of the number of different types of all non-volatile metabolites.

A total of 138 metabolites were detected and annotated from LSCKs based on LC-MS/MS untargeted metabolomics (Supplementary Table S1). Among which, a total of 124 metabolites were classified in 13 groups including more than 1 metabolites, as shown in Figure 3. The 13 groups were amino acids, peptides, and analogues (29, 23.39%), fatty acyls (19, 15.32%), organic acids and derivatives (14, 11.29%), carbohydrates and carbohydrate conjugates (15, 12.10%), benzene and substituted derivatives (10, 8.06%), organooxygen compounds (7, 5.65%), imidazopyrimidines (6, 4.84%), phenols (4, 3.23%), pyridines and derivatives (3, 2.42%), diazines (3, 2.42%), pyrimidine nucleosides (2, 1.61%), steroids and steroid derivatives (2, 1.61%), and others (10, 8.06%). According to the compositions and contents of metabolites, non-volatile metabolites in the first five groups were considered the major metabolites in LSCKs. Amino acids, peptides, and analogues accounted for the highest proportions in LSCKs. Amino acids, strongly associated with food taste and odor, are the main contributors to fermented foods flavors (11). A variety of amino acids, including L-glutamic acid, L-aspartic acid, L-glutamine, L-serine, L-phenylalanine, L-cysteine, L-leucine, L-valine, and others, were detected in the LSCKs. Free amino acids play different roles in the composition of fermented foods flavors. Alanine and arginine mainly provide sweetness accompanied by a monosodium-like taste, while glutamic acid and aspartic acid are the main contributors to the freshness of fermented foods, and tryptophan and phenylalanine affect astringent and bitter tastes of foods (12). During the fermentation of LSCK, microorganisms could utilize nutrients to synthesize various types of free amino acids, providing the fermented LSCK characteristic flavor.

### 3.4 Classification and identification of differential non-volatile metabolites in LSCKs

**Figure 4:**
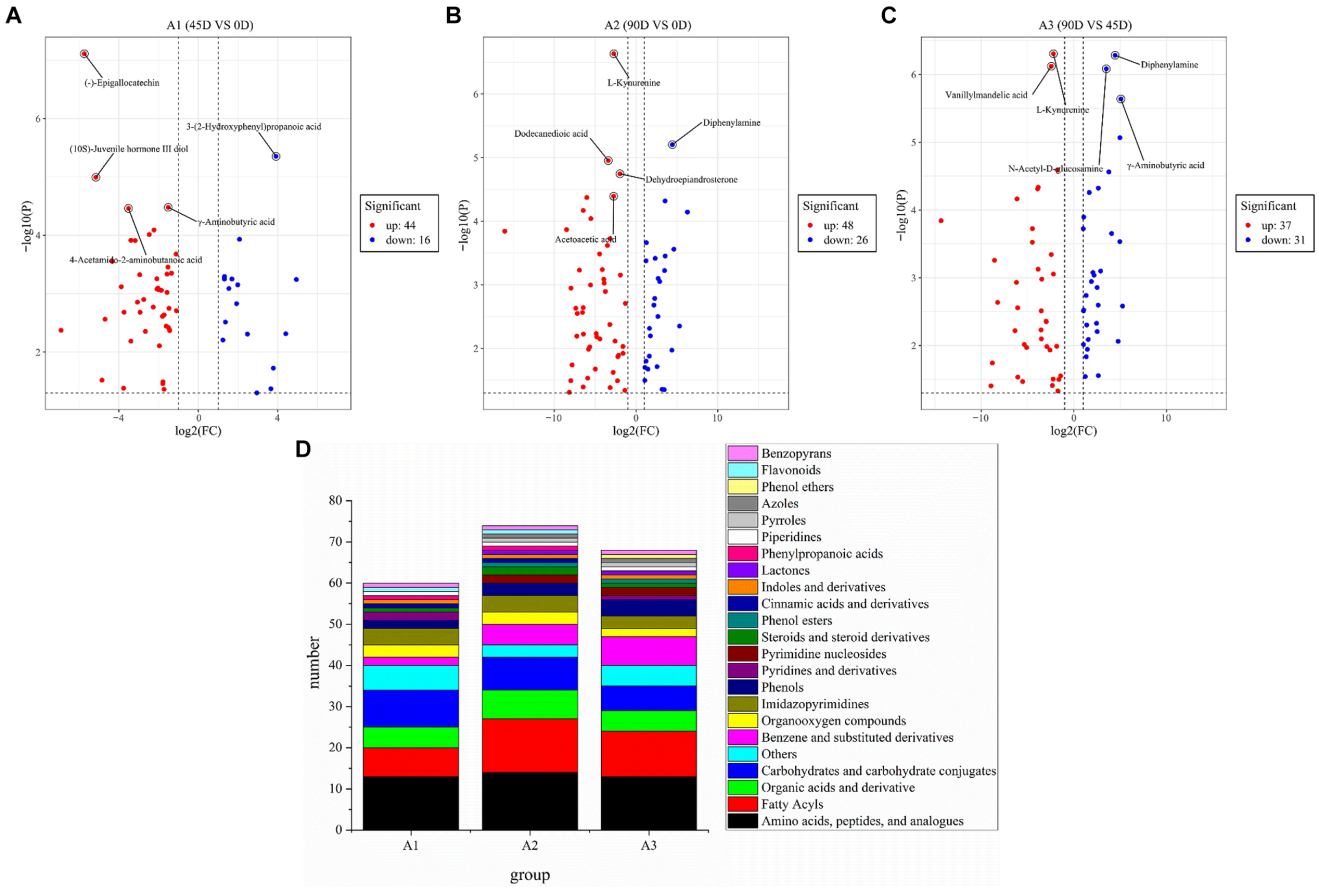
Volcano plot of the differential non-volatile metabolites of 0D–45D **(A)**, 0D–90D **(B)**, 45D–90D **(C)** and classification information for differential metabolites of LSCK **(D)**.

In order to further investigate the differences in non-volatile metabolites of LSCKs, 60, 74, and 68 differential metabolites were, respectively, screened from the three treatment groups of A1 (0D–45D), A2 (0D–90D), and A3 (45D–90D) (*p* < 0.05, VIP >1, and Log2FC >1), as shown in Figure 4. Five most significant differential metabolites were marked in Figures 4A–C. In A1 (0D–45D) group, the differential metabolites (−)-epigallocatechin, (10S)-juvenile hormone III diol, *γ*-aminobutyric acid, and 4-acetamido-2-aminobutanoic acid were up-regulated, while 3-(2-hydroxyphenyl)propanoic acid was down-regulated. In A2 (0D–90D) group, L-kynurenine, dodecanedioic acid, dehydroepiandrosterone, and acetoacetic acid were up-regulated, whereas diphenylamine was down-regulated. However, in A3 (45D–90D) group, the two differential metabolites vanillylmandelic acid and L-kynurenine were up-regulated, and three differential metabolites diphenylamine, N-acetyl-D-glucosamine, and *γ*-aminobutyric acid were down-regulated. Throughout the fermentation process, the number of up-regulated metabolites exceeded that of downregulated ones, indicating an overall increase in metabolite levels. This increase suggests enhanced synthesis of flavor precursor substances and flavor compounds (13).

**Figure 5:**
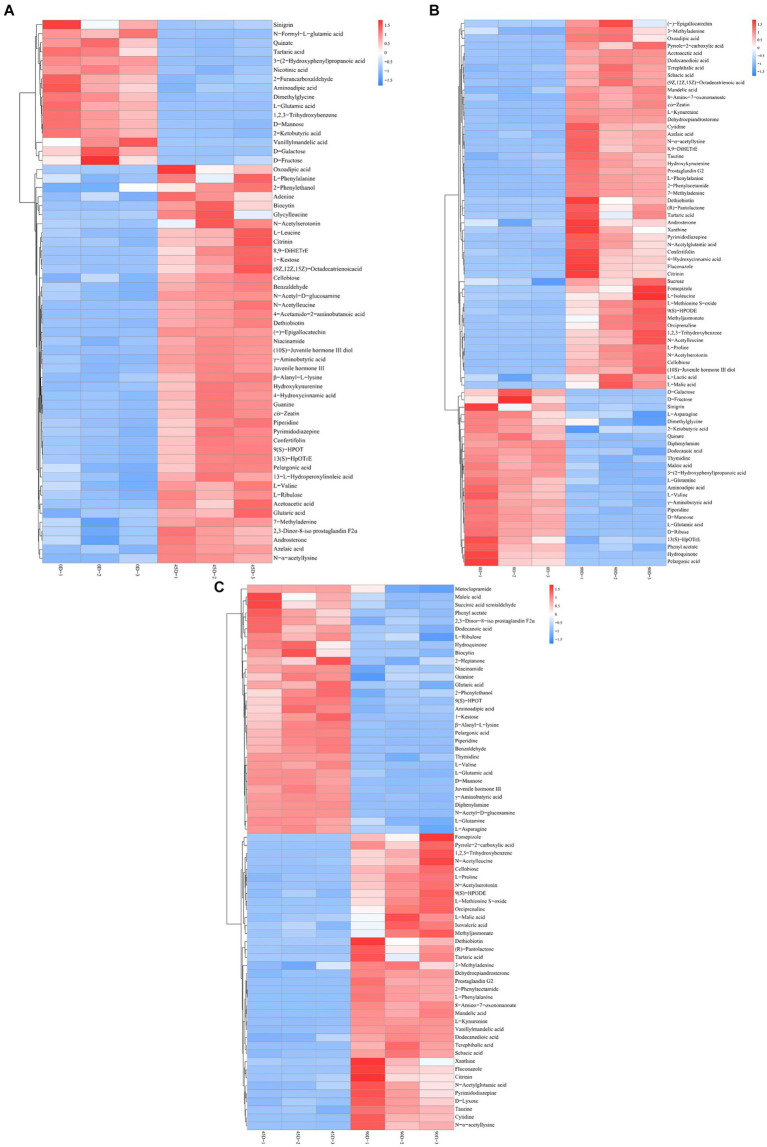
Hierarchical cluster analysis of main differential metabolites in LSCK, **(A–C)** indicated the differential metabolites of groups A1, A2, and A3 respectively.

Amino acids, peptides and analogs, fatty acyls, organic acids and derivatives, and carbohydrates and carbohydrate conjugates were the common major differential metabolites in the three LSCKs, as shown in Figure 4D and Supplementary Table S2. During LSCK fermentation, microorganisms metabolize nutrients such as sugars, fats, and proteins through enzymatic reactions, producing a variety of metabolites such as organic acids, amino acids, and fatty acids (14). Figures 5A–C show the changes in differential metabolites in groups A1 (0D–45D), A2 (0D–90D), and A3 (45D–90D), respectively.

#### 3.4.1 Amino acids, peptides, and analogues in LSCKs

Amino acids, peptides, and analogues accounted for the largest proportion of all differential metabolites. Compared to 0D, 13 and 14 amino acids, peptides, and analogues showed significant differences at 45D (9 up-regulated and 4 down-regulated) and 90D (7 up-regulated and 7 down-regulated), respectively. Furthermore, 90D showed different trends in amino acid differential metabolites compared with 45D (6 up-regulated and 7 down-regulated). Amino acid metabolism is a key factor influencing fermented foods flavors (15). Alanine and L-aspartic acid, which provide sweetness and freshness, were produced in the highest amount at 90D (90D > 45D > 0D), and these free amino acids positively contributed to the sensory properties of kohlrabi (16). Similarly, the two essential amino acids L-phenylalanine and L-isoleucine increased progressively with fermentation time. Microorganisms biosynthesize biocatalysts such as cellulases and proteases during fermentation, which degrade kohlrabi cell walls, decompose proteins, and release free amino acids (12). Moreover, peptides and proteins can also be metabolized by microorganisms, producing various amino acids (17). On the other hand, some amino acids, such as L-glutamic acid, L-asparagine, and aminoadipic acid, decreased during LSCK fermentation, likely due to their utilization by microorganisms. For example, lactic acid bacteria (LAB) can metabolize amino acids to produce flavor substances such as phenyl lactic acid, phenyl acetate, and phenylethanol (18). Interestingly, L-valine was the only essential amino acid decreased during the later stage of fermentation, with the lowest levels observed at 90D. L-valine serves as a nitrogen source for yeast (19), which is probably utilized by yeast during fermentation. As a bitter amino acid, the decrease of valine is beneficial to LSCK flavor (20). The decrease of branched chain amino acid like L-valine and L-leucine in the later fermentation stage was probably due to their metabolism by LAB and yeast, producing branched aldehydes and alcohols, which contributed greatly to the unique flavor of fermented foods (21). Therefore, the degradation and release of free amino acids occur simultaneously during microbial fermentation in LSCK, which was consistent with a previous study (22). Amino acids not only greatly enhance flavor but are also key bioactive compounds that dominate various life activities in plants. They are involved in the synthesis of amines, proteins, alkaloids, enzymes, vitamins, terpenoids, purines, and pyrimidines. Additionally, amino acids are essential for plant stress defense and reducing abiotic stresses (23).

#### 3.4.2 Fatty acyls

Most of the fatty acyls increased during LSCKs fermentation. Compared to 0D, 7 fatty acyls in 45D were up-regulated. While, 10 fatty acyls were up-regulated and 3 fatty acyls were down-regulated in 90D, compared to 0D. Eleven differential fatty acyls were detected in 90D and 45D, among which 8 fatty acyls decreased in 45D. Most of the up-regulated fatty acyls were unsaturated fatty acid during fermentation. Unsaturated fatty acids are a class of essential fatty acids with various biological functions, such as promoting human growth and development, maintaining cellular homeostasis, and lowering blood pressure and lipids (24). (9Z,12Z,15Z)-Octadecatrienoic acid, also known as α-linolenic acid, has multiple functions, including improving cardiovascular health, enhancing immunity, and providing anti-inflammatory effects (25). During kohlrabi fermentation, *Lactobacillus plantarum* and *Bifidobacterium* metabolize fats to produce α-linolenic acid and convert it into longer-chain ω-3 fatty acids like eicosapentaenoic acid and docosahexaenoic acid (26). The content of α-linolenic acid in 45D and 90D was 10.47 and 17.48 times higher than that of 0D, respectively. The increase in medium-chain fatty acids like pelargonic acid, dodecanoic acid, heptanoic acid, and isovaleric acid is correlated with some biosynthesis pathways. Microorganisms such as *Lactobacilli*, *Lactococcus* spp., and *Streptococcus* spp., with lipolytic enzymes, also participate in the formation of medium-chain fatty acids (27). The content of some fatty acids, such as pelargonic acid and dodecanoic acid, decreased significantly in the later stage of fermentation (45–90 days), likely due to microbial metabolic activity and membrane lipid degradation (28). Pelargonic acid and dethiobiotin were differential fatty acyl metabolites present in all three groups (A0, A1 and A2). Pelargonic acid, a saturated fatty acid with a putrid and irritating odor, decreased significantly in the later stage of fermentation (45–90 days), resulting in the lowest levels in 90D and promoting positive flavor formation in LSCK. Dethiobiotin is a medium-chain fatty acid widely used in biomedical applications as a precursor for synthesizing biotin (29). The described changes in differential fatty acyl metabolites positively influenced the flavor improvement and nutrient enrichment of LSCKs.

#### 3.4.3 Organic acids and derivatives

The compositions and contents of organic acids play a critical role in fermented vegetables flavors. Most of the organic acids significantly increased in the earlier fermentation stage (0–45 days) and decreased in the later stage (45–90 days). It has been documented that low-salt fermented foods with relatively higher water activity may promote the growth of LAB (30), which could affect the production of organic acids and thus affect the taste and acceptability of LSCK. During fermentation, microorganisms utilize nutrients such as glucose, sucrose, and fructose to produce organic acids through metabolic pathways, including glycolysis, the pentose phosphate pathway, and the citric acid cycle (TCA cycle). This metabolic activity changes the pH of the fermentation environment, contributing to the characteristic flavor of fermented kohlrabi and reducing contamination by spoilage microorganisms (31). The decline in organic acids during the late fermentation stage (45–90 days) can be attributed to various factors. Some studies have suggested that microorganisms prioritize organic acids as a carbon source in the later stages of vegetable fermentation due to the depletion of nutrients such as carbohydrates and proteins, and the accumulation of total acids during fermentation leads to a decrease in pH, which affects microbial reproduction and metabolism (32). Lactic acid was a differential metabolite in group A2 (0D–90D), which continuously increased during fermentation. Lactic acid is the most common core organic acid in fermented vegetables, usually produced by *Lactobacillus*, and contributes greatly to many fermented foods flavors. The increase in lactic acid during fermentation is mainly dependent on the Embden-Meyerhof pathway (EMP) as well as the degradation of other organic acids (e.g., malic and citric acid) (33). Pyruvate, an essential intermediate product in basic metabolic pathways such as glycolysis, malate-lactate fermentation, and the TCA cycle, increased in the early stage (0–45 days) and decreased in the later stage (45–90 days). Environmental limitations (pH and oxygen content) reduce the rate of pyruvate production during the later stage of fermentation. Under anaerobic conditions, pyruvate produced from glycolysis is ultimately converted to lactic acid through lactic acid fermentation (34). As a key substance in the central carbon metabolic pathway, pyruvate is also consumed in metabolic pathways such as the TCA cycle and branched-chain amino acid synthesis (35). Malic acid was the differential metabolite between group A2 (0D–90D) and group A3 (45D–90D) and gradually increased during fermentation. With its strong acidity, malic acid directly enhances the sourness of fermented foods (36). Organic acids are also essential in regulating plant vital activities. For instance, salicylic acid plays a primary role in plant defense and immune responses (37), while benzene-ring-containing carboxylic acids and their derivatives can induce and produce resistance in a wide variety of plants (38). The production of organic acids during kohlrabi fermentation involves many complex metabolic networks and enzyme-catalyzed reactions, necessitating further investigation into the flavor composition and related metabolic pathways of fermented kohlrabi.

#### 3.4.4 Carbohydrates and carbohydrate conjugates

Most of the carbohydrates and carbohydrate conjugates declined with the extension of fermentation period. For example, differential metabolites such as D-glucose, D-fructose, D-galactose, and D-ribose decreased continuously, reaching their lowest in 90D. Carbohydrate metabolism is fundamental to microbial growth and the formation of flavor compounds. Guérin et al. (39) reported that the characteristic sweetness of fermented products was largely dependent on the release of monosaccharides, especially glucose, fructose, and galactose. It is well known that *Lactobacillus lactis* utilizes straight-chain starch and sucrose to release D-glucose, which is finally metabolized to produce pyruvic acid and acetyl-coenzyme A via the EMP pathway. Acetyl-coenzyme A is a substrate for the further production of flavors (e.g., organic acids and ethanol) (40). The sucrose content in LSCK did not change remarkably during fermentation since the molecular structure of sucrose was more complex compared to other monosaccharides, requiring multiple metabolic steps to be fully utilized by microorganisms. Microorganisms primarily metabolize glucose and fructose in the early fermentation stages, while sucrose utilization remains relatively low, consistent with a previous study (41). Similar to sucrose, cellobiose, a disaccharide composed of two glucose molecules produced by the enzymatic hydrolysis of lignocellulose or cellulose (42), significantly increased among the three treatment groups (A1, A2, and A3) during fermentation. This increase may be related to the abundant dietary fiber in kohlrabi (43). D-mannose, another common differential metabolite among the three groups (A1, A2, and A3), declined significantly during fermentation, likely due to its metabolism by yeasts in LSCK (44).

### 3.5 Common differential metabolites in LSCKs

**Figure 6:**
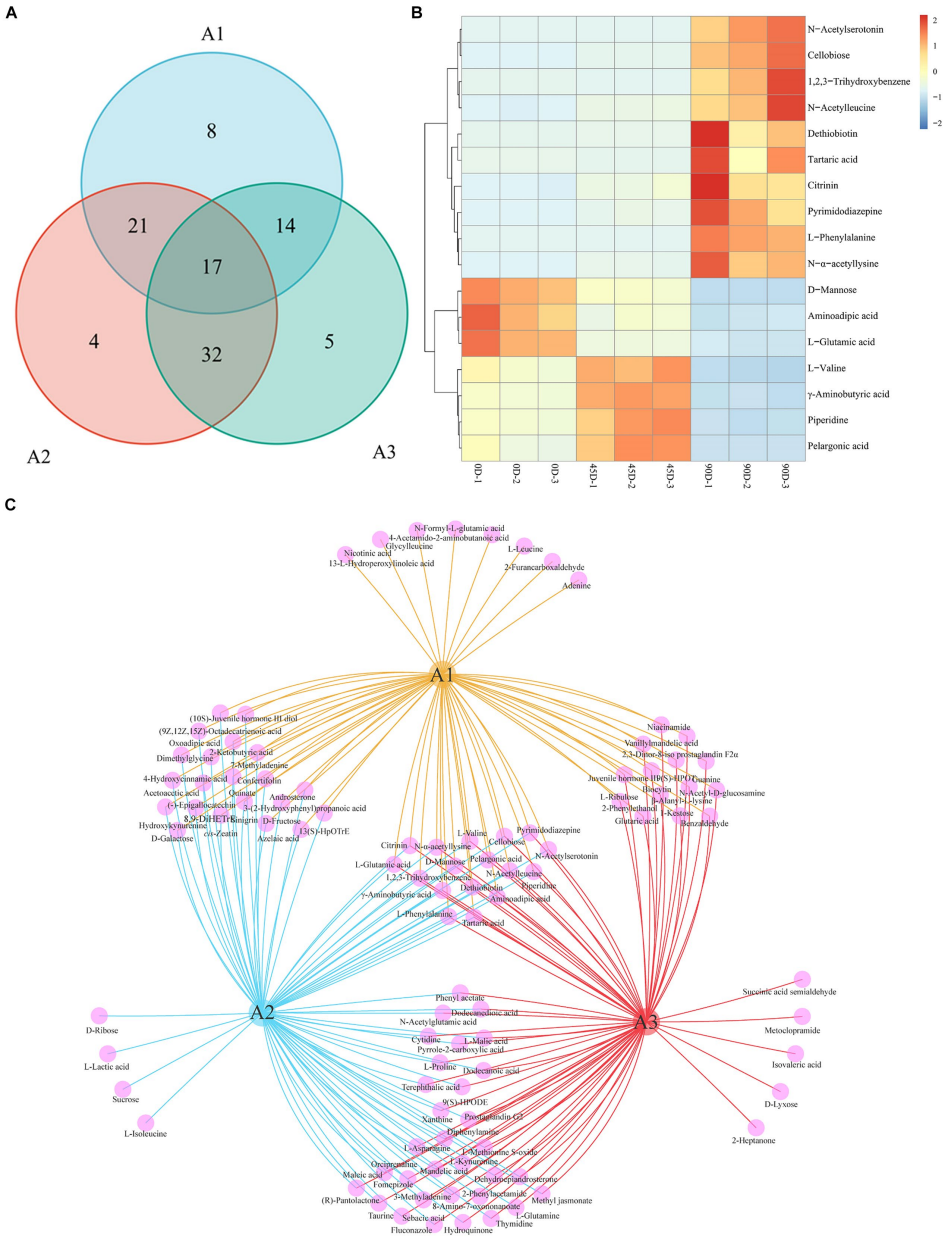
Venn diagram and common differential metabolites analysis for the three treatment groups of LSCK. **(A)** The number of metabolic sets of Venn diagram. **(B)** Hierarchical clustering heatmap of common differential metabolites. **(C)** Venn diagram visualization results.

Venn diagrams were constructed for statistically analyzing the differential metabolites in the three groups (A1, A2, and A3), as shown in Figures 6A,C. The three treatment groups totally shared 17 common metabolites (Figure 6B), including 7 amino acids, peptides, and analogues (L-phenylalanine, aminoadipic acid, L-valine, L-glutamic acid, N-alpha-acetyllysine, N-acetylleucine, and *γ*-aminobutyric acid), 3 carbohydrates and carbohydrate conjugates (tartaric acid, cellobiose, and D-mannose), 2 fatty acyls (pelargonic acid and dethiobiotin), 1 benzopyrans (citrinin), 1 indoles and derivative (N-acetylserotonin), 1 phenol (1,2,3-trihydroxybenzene), 1 piperidine (piperidine), and 1 other (pyrimidodiazepine). It was obvious that these 17 differential metabolites varied significantly, which could be used as potential biomarkers for distinguishing the three different LSCKs. The 0D produced the highest levels of D-mannose, aminoadipic acid, and L-glutamic acid, which decreased significantly as fermentation progressed. L-Glutamic acid, an amino acid that provides fresh flavor, can be converted to other amino acids through various biochemical pathways during fermentation. For example, glutamic acid can react with α-ketoglutarate to form alanine via glutamic oxaloacetic transaminase (GOT) in the alanine, aspartate, and glutamate metabolism pathway. Additionally, a portion of glutamate can also be converted to aspartic acid, catalyzed by GOT, leading to a significant increase in aspartic acid content during LSCK fermentation (45). The 45D had higher differential metabolites of L-valine, *γ*-aminobutyric acid, piperidine, and pelargonic acid. L-valine and *γ*-aminobutyric acid, categorized as amino acids, peptides, and analogues, initially increased and then decreased during LSCK fermentation. This increase during the early stages might be due to protein degradation in LSCK, while the subsequent decrease could result from protein synthesis and participation in other metabolic processes, consistent with a previous studies (46). In 90D, the most abundant differential metabolites were N-acetylserotonin, cellobiose, 1,2,3-trihydroxybenzene, N-acetylleucine, dethiobiotin, tartaric acid, citrinin, pyrimidodiazepine, L-phenylalanine, and N-α-acetyllysine. Tartaric acid with an excellent antioxidant property gradually increased during fermentation, potentially enhancing the antioxidant activity of LSCK (47).

### 3.6 KEGG enrichment and metabolic pathway analysis

**Figure 7:**
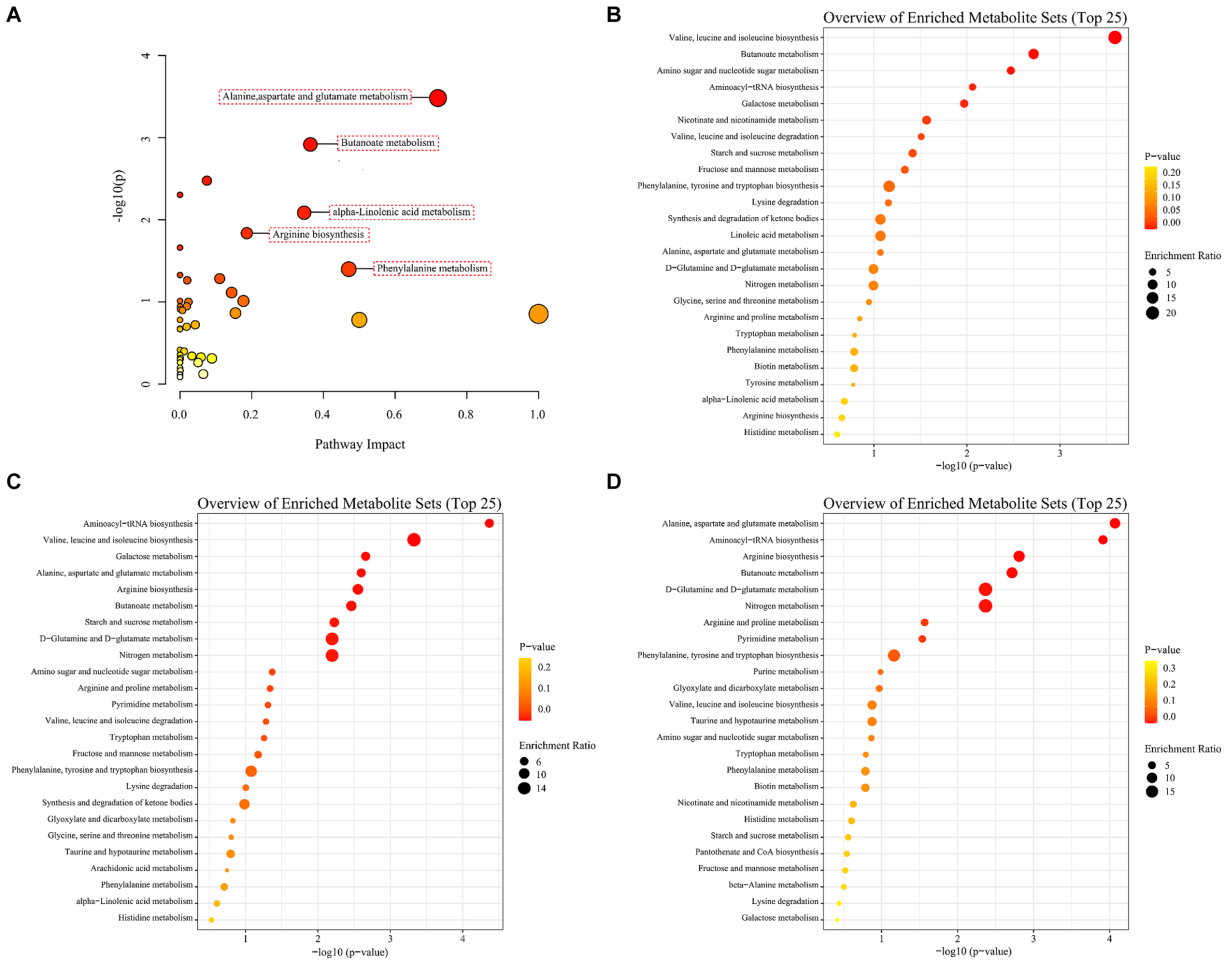
Enrichment analysis of metabolic pathways of differential metabolites in LSCK, **(A)** all differential metabolites; **(B)** 0D and 45D; **(C)** 0D and 90D; **(D)** 45D and 90D.

To investigate the disturbed metabolic pathways, pathway enrichment analysis on all the differential non-volatile metabolites was performed using the KEGG database [−log10(*p*)>1.3, impact >0.1]. As shown in Figure 7A, the most enriched pathways were alanine, aspartate and glutamate metabolism (impact = 0.72), butanoate metabolism (impact = 0.36), α-linolenic acid metabolism (impact = 0.35), arginine biosynthesis (impact = 0.19), and phenylalanine metabolism (impact = 0.47). However, the KEGG enrichment pathways were different among the A1 (0D–45D), A2 (0D–90D), and A3 (45D–90D) groups. As for A1 (0D–45D), the top five pathways were valine, leucine and isoleucine biosynthesis, butanoate metabolism, amino sugar and nucleotide sugar metabolism, aminoacyl-tRNA biosynthesis, and galactose metabolism (Figure 7B). For the A2 (0D–90D) group, the top five pathways were aminoacyl-tRNA biosynthesis, valine, leucine, and isoleucine biosynthesis, galactose metabolism, alanine, aspartate and glutamate metabolism, and arginine biosynthesis (Figure 7C). While for the A3 (45D–90D) group, the primary pathways were alanine, aspartate and glutamate metabolism, aminoacyl-tRNA biosynthesis, arginine biosynthesis, butanoate metabolism, and D-glutamine and D-glutamate metabolism (Figure 7D). These results suggested that the main metabolic pathways in LSCKs changed with the fermentation period, resulting in differential metabolites in LSCKs. Consequently, the metabolic processes need to be further investigated to more accurately monitor the flavor changes of LSCKs during fermentation.

**Figure 8:**
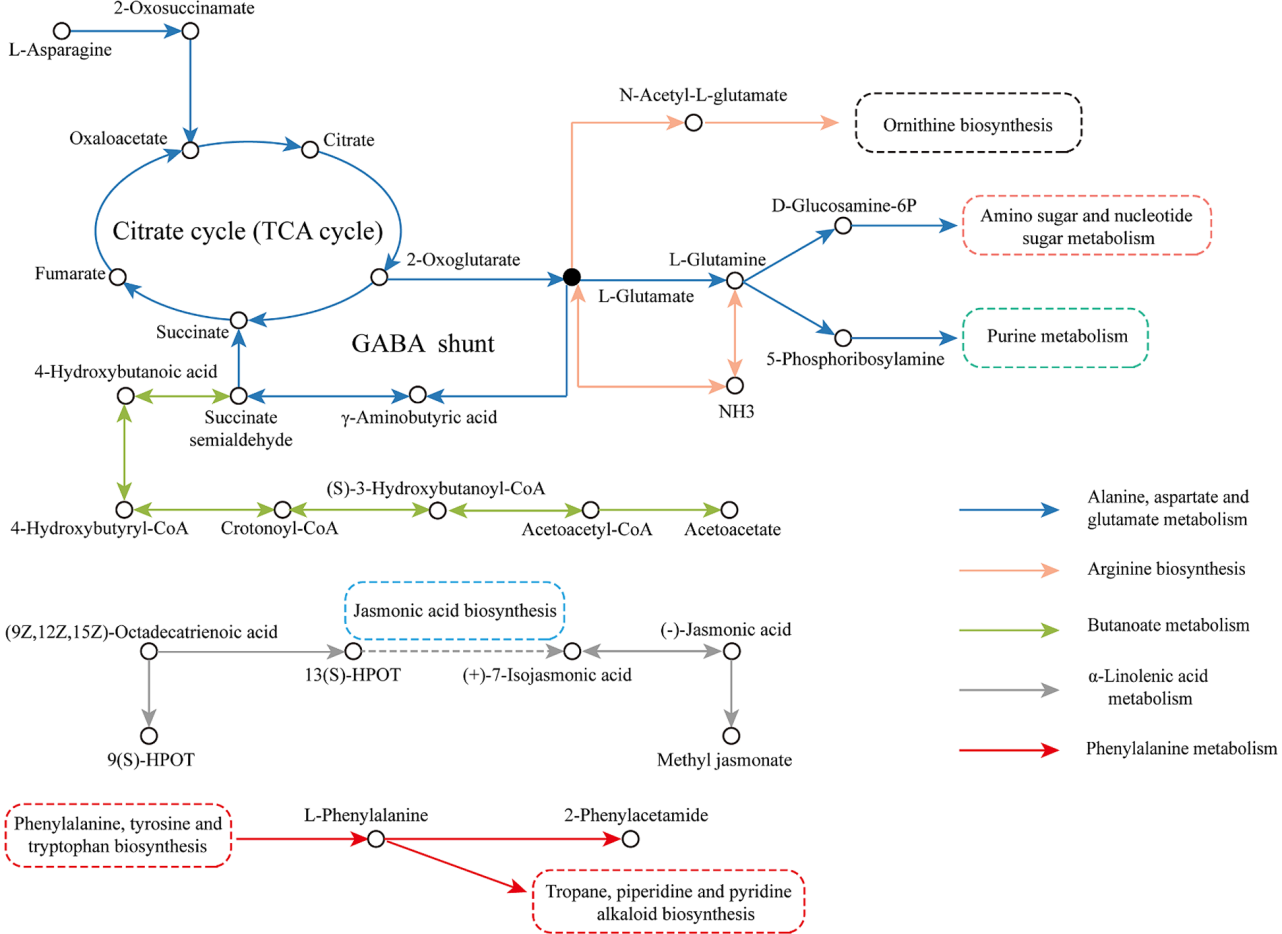
The major differential metabolic pathways in LSCK during fermentation.

To better analyze the transformation of metabolites during LSCK fermentation, the five important metabolic pathways were integrated into a metabolic network, as seen in Figure 8. In the alanine, aspartate and glutamate metabolism pathway, the L-asparagine reacted with asparagine-oxo-acid transaminase to produce 2-oxosuccinamate, which was then converted to oxaloacetate catalyzed by *ω*-amidase and subsequently entered the TCA cycle. 2-oxoglutarate was a product of the TCA cycle, which generated L-glutamate in the presence of glutamate synthase (NADH). Some of the L-glutamate could produce succinate through the *γ*-aminobutyrate shunt pathway and re-entered the TCA cycle. Part of the 2-oxoglutarate was converted to L-glutamine and catalyzed by phosphate aminotransferase and amidophosphoribosyl transferase to produce D-glucosamine 6-phosphate and 5-phosphoribosylamine, which participated in amino sugar and nucleotide sugar metabolism and purine metabolism, respectively. In the butanoate metabolism pathway, the L-glutamate generated by alanine, aspartate and glutamate metabolism was used as the raw material to produce the succinate semialdehyde through the amino acid metabolism pathway. Succinate semialdehyde was then reduced to 4-hydroxybutanoic acid by glyoxylate reductase and reacted with CoA ligase (ADP-forming) to form 4-hydroxybutyryl-CoA, which generated crotonoyl-CoA catalyzed by 4-hydroxybutanoyl-CoA dehydratase. Crotonoyl-CoA was then transformed to (S)-3-hydroxybutanoyl-CoA catalyzed by enoyl-CoA hydratase, which finally entered the ketone body biosynthesis pathway to produce the final product acetoacetate. In the arginine biosynthesis pathway, 2-oxoglutarate produced by TCA cycle was used as a raw material to generate L-glutamate. A part of L-glutamate was catalyzed by glutamate dehydrogenase (NADP+) to generate NH3, which was then converted to L-glutamine under the catalysis of glutamine synthetase. The other L-glutamate was catalyzed by glutamate N-acetyltransferase/amino-acid N-acetyltransferase to synthesize N-acetyl-L-glutamate and entered the ornithine biosynthesis. In the α-linolenic acid metabolism pathway, α-linolenic acid was used to generate 9(S)-HPOT and 13(S)-HPOT under the catalysis of lipoxygenase. 13(S)-HPOT entered the jasmonic acid biosynthesis pathway to produce (+)-7-isojasmonic acid, which was subsequently converted to (−)-jasmonic acid, and produce the final product methyl jasmonate catalyzed by the jasmonate O-methyltransferase. In the phenylalanine metabolism pathway, L-phenylalanine produced by phenylalanine, tyrosine and tryptophan biosynthesis was utilized as the raw material to produce 2-phenylacetamide catalyzed by L-phenylalanine oxidase. While, some of the L-phenylalanine directly participated in the tropane, piperidine and pyridine alkaloid biosynthesis.

L-glutamic acid is central to amino acid metabolism. Besides the described pathways above, L-glutamic acid is also involved in the arginine and proline metabolism, histidine metabolism, D-amino acid metabolism, glutathione metabolism, ornithine biosynthesis, and other important metabolic pathways. In the butanoate metabolism pathway, L-glutamic acid is converted to *γ*-amino butyric acid (GABA), a four-carbon nonprotein amino acid, which is a major neurotransmitter in the mammalian central nervous system. GABA-rich foods exhibit a variety of pharmacological functions, such as antihypertensive and antidepressant (48). GABA levels increased significantly in the earlier fermentation period (0–45 days) and decreased remarkably in the later fermentation period (45–90 days), which was probably due to the glutamate decarboxylation reaction catalyzed by the enzyme glutamate decarboxylase, produced by microorganisms. This enzyme exhibits relatively higher activity at a low pH condition. Microorganisms such as *Lactobacillus lactis* in the earlier fermentation period produce a large number of organic acids, enhancing GABA shunt activity. However, in the later stages, some of the organic acids might be consumed as a carbon source, while GABA was also used to synthesize acetoacetate, an intermediate product in the butanoate metabolism pathway (49). Methyl jasmonate, the final product in the α-linolenic acid metabolism pathway, increased significantly during fermentation. Methyl jasmonate can activate α-linolenic acid metabolism, raising α-linolenic acid concentration and enhancing the activities of lipoxygenase, allene oxide synthase, and allene oxide cyclase (50).

## 4 Conclusion

In this study, the non-volatile metabolites of a novel low-salt kohlrabies industrially fermented for 0 day, 45 days, and 90 days were analyzed by LC-MS/MS coupled with multivariate statistical analysis. A total of 202 differential non-volatile metabolites were identified among the A1 (0D–45D), A2 (0D–90D), and A3 (45D–90D) (VIP >1, *p* < 0.05, Log2FC >1). The differential non-volatile metabolites were mainly amino acids, peptides, and analogues, fatty acyls, organic acids and derivatives, and carbohydrates and carbohydrate conjugates. Furthermore, a total of 17 major differential non-volatile metabolites were screened based on Venn diagrams analysis, and a total of five relevant metabolic pathways were obtained from metabolic pathway analysis for the first time. Optimization of the LSCK processing and utilization of nutritional and functional non-volatile metabolites in LSCK will be investigated in the future. The microbial diversity and succession will be investigated in the future as well as the mechanisms on the formation of the non-volatile metabolites in LSCK regulated by microorganisms through metagenomics and other techniques. This work reveals the non-volatile metabolites of LSCK by LC-MS/MS-based metabolomics for the first time and provides a theoretical basis for flavor regulation of the industrially fermented LSCK.

## Data availability statement

The original contributions presented in the study are included in the article/Supplementary material, further inquiries can be directed to the corresponding authors.

## Author contributions

XJ: Formal analysis, Methodology, Writing – original draft. XW: Writing – original draft. HC: Investigation, Writing – original draft. DL: Methodology, Writing – original draft. BD: Formal analysis, Writing – original draft. LA: Formal analysis, Writing – original draft. JY: Formal analysis, Writing – original draft. XN: Data curation, Formal analysis, Supervision, Writing – review & editing. ZZ: Conceptualization, Data curation, Formal analysis, Funding acquisition, Methodology, Validation, Visualization, Writing – review & editing.

## Footnotes

^1^ http://www.hmdb.ca/

^2^ https://metlin.scripps.edu/

^3^ http://www.genome.jp/kegg/

^4^ https://docs.scipy.org/doc/scipy/
