# Supplementary material for: Non-targeted metabolomic analysis of non-volatile metabolites in a novel Chinese industrially fermented low-salt kohlrabi
Source: Front Nutr. 2024 Aug 30;11:1450789. doi: 10.3389/fnut.2024.1450789 (PMC11397298; doi:10.3389/fnut.2024.1450789)
Supplement: Supplementary file 1 [file Table_1.docx]

Supplementary Material

# Supplementary Data

Table S1. Metabolite peak ratio of kohlrabi in 0D, 45D and 90D groups

| Name | KEGG | Type | D0 | D45 | D90 |
| --- | --- | --- | --- | --- | --- |
| S-Adenosylmethionine | C00019 | [M]+ | 64130481.08±8005812.52 | 67190443.75±6792585.99 | 79023036.93±53237745.67 |
| Pyruvic acid | C00022 | [M+H]+ | 51460746.35±67840720.9 | 74186984.97±63297549.4 | 2720112.16±1472880.36 |
| L-Glutamic acid | C00025 | [M+H]+ | 2574212032.42±353022951.94 | 648166298.36±17823103.23 | 104347509.27±46930036.04 |
| L-Aspartic acid | C00049 | [M+H]+ | 120414350.3±4325578.96 | 89188278.53±7083891.62 | 251527991.09±200801746.69 |
| L-Glutamine | C00064 | [M+H]+ | 7779566624.3±1170498391.18 | 6352018951.3±321086937.85 | 1706593598.03±910925901.45 |
| L-Serine | C00065 | [M+H]+ | 34731133.9±5848317.93 | 41517119.61±37162770.27 | 27510388.9±1729940.91 |
| L-Phenylalanine | C00079 | [M-H2O-H]- | 966.48±510.56 | 3196.47±1221.26 | 65905698.77±8050599.37 |
| L-Cysteine | C00097 | [M]+ | 58773517.5±3342886.9 | 31120072.79±1840727.21 | 46984490.05±19741042.09 |
| β-Alanine | C00099 | [M+H]+ | 236.81±0 | 6174824.68±5921883.94 | 76400121.95±55055003.61 |
| 2-Ketobutyric acid | C00109 | [M-H]- | 503928166.22±49149310.34 | 201430164.24±13701963.76 | 150000566.24±106219692.15 |
| Choline | C00114 | [M+H]+ | 178418964.26±259689247.03 | 5784885.32±2798363.48 | 1858840068.73±2071643526.74 |
| D-Ribose | C00121 | [M-H]- | 168609303.02±15324786.15 | 95208000.29±3084538.13 | 72551356.69±1743720.08 |
| L-Leucine | C00123 | [M+H]+ | 8032781.75±1290499.21 | 22033049.18±3888138.67 | 10735352.26±7811755.79 |
| N-Acetyl-D-glucosamine | C00140 | [M+H]+ | 3736050.77±3455987.57 | 33734794.71±681606.5 | 2980184.66±766715.38 |
| Adenine | C00147 | [M-H]- | 1722216.48±951181.13 | 6675585.11±1452673.92 | 21776893.16±15975740.05 |
| L-Proline | C00148 | [M+H]+ | 39684793.88±12612086.54 | 55463156.55±32754330.59 | 611389857.71±108347263.8 |
| L-Malic acid | C00149 | [M-H]- | 5837194.2±338739.62 | 10021429.26±1159276.96 | 27176766.85±8747570.48 |
| L-Asparagine | C00152 | [M+H]+ | 128232350.46±23168199.07 | 187191612.73±10107819.64 | 41459263.01±26786738.78 |
| Niacinamide | C00153 | [M+H]+ | 47847643.46±5470246.3 | 103554891.95±5117018.05 | 24732029.26±14252769.51 |
| D-Mannose | C00159 | [M-H]- | 4791103213.13±484398635.8 | 1920197585.32±110961452.33 | 61230349.84±11338008.33 |
| Acetoacetic acid | C00164 | [M+H-H2O]+ | 50937870.12±8388169.97 | 678862391.23±152853971.59 | 340601228.87±24283971.3 |
| Thymine | C00178 | [M]+ | 63279740.41±40836199.62 | 34670152.33±27699822.5 | 138482719.27±108801851.13 |
| L-Valine | C00183 | [M-H]- | 104579183.89±23729372.2 | 224444632.92±16245818.61 | 16378521.36±3919938.17 |
| Cellobiose | C00185 | [M-H2O-H]- | 429968.24±542572.76 | 3588162.3±431398.24 | 52079629.85±9032714.12 |
| D-Glucuronic Acid | C00191 | [M-H]- | 2087802.66±1733408.43 | 47435196.65±82160113.88 | 19328204.51±14439734 |
| Adenosine | C00212 | [M-H]- | 3648462.76±3282156.39 | 2389726.16±1448795.85 | 2799869.66±1781443.45 |
| Thymidine | C00214 | [M]- | 12851942.97±737786.75 | 11262290.93±38654.06 | 5381110.03±695295.07 |
| Acetylphosphate | C00227 | [M]+ | 12609397.93±6219601.95 | 397432182.95±403821080.83 | 127526951.39±74245604.79 |
| Succinic acid semialdehyde | C00232 | [M+H]+ | 174612538.81±302437378.68 | 219967957.11±53357181.82 | 87343673.37±16254172.75 |
| Ketoleucine | C00233 | [M+H-H2O]+ | 17490810.43±5109928.79 | 13139017.82±7456389.71 | 18012807.62±11226365.74 |
| Guanine | C00242 | [M-H]- | 29833070.14±1893213.25 | 88613549.32±11512582.8 | 30169704.91±17238116.64 |
| Taurine | C00245 | [M-H]- | 11292417.81±1644920.58 | 6065286.03±2200661.17 | 27895277.66±3636068.08 |
| Nicotinic acid | C00253 | [M-H]- | 111071.2±7580.39 | 26266.57±6254.63 | 5286894.74±5105405.81 |
| Benzaldehyde | C00261 | [M+H]+ | 26387636.48±4985217.6 | 67125097.76±4387499.02 | 33305554.32±600781.75 |
| Quinate | C00296 | [M-H]- | 604043765.32±97261774.67 | 158569371.63±20105693.05 | 81370125.91±30562970.55 |
| L-Ribulose | C00310 | [M+H-H2O]+ | 20886687.83±4495685.05 | 62049865.59±5069087.79 | 23799116.44±10676270.28 |
| Oxoadipic acid | C00322 | [M-H]- | 5493851.29±966043.29 | 158152111.89±80402093.96 | 118099944.55±38381490.36 |
| L-Kynurenine | C00328 | [M+H]+ | 7968076±469956.24 | 11405644.61±708848.88 | 52496412.91±975673.49 |
| γ-Aminobutyric acid | C00334 | [M+H]+ | 1096221661.48±133867998.33 | 3123578728.69±106149468.46 | 92813374.73±76254339.04 |
| *cis*-Zeatin | C00371 | [M+H-H2O]+ | 413902.35±43270.82 | 1750872.03±226829.82 | 27024613.2±2386319.07 |
| Xanthine | C00385 | [M+H]+ | 29017666.76±3875211.05 | 42250999.68±1955131.48 | 139950184.61±59574862.17 |
| L-Isoleucine | C00407 | [M+H]+ | 13268055.14±3967640.48 | 25459688.69±3452101.48 | 119650746.03±61984187.62 |
| trans-Cinnamate | C00423 | [M-H]- | 3714859.64±761594.1 | 7245378.79±1029208.21 | 101880727.17±109313337.16 |
| Dihydrouracil | C00429 | [M-H]- | 18822310.76±18312605.27 | 13986978.63±835005.26 | 154519902.07±113120909.13 |
| 5-Aminopentanoic acid | C00431 | [M]- | 26727740.84±22698342.62 | 17397173.54±4919385.2 | 27264304.28±30037656.23 |
| Cytidine | C00475 | [M]- | 4093758.24±672331.88 | 5735999.96±3134664.71 | 384818544.91±99836303.01 |
| D-Lyxose | C00476 | [M+H]+ | 19770170.31±12247427.94 | 4869483.58±2586260.61 | 38906591.4±9817184.64 |
| Glutaric acid | C00489 | [M]+ | 53227722.78±15643631.75 | 175021376.86±26233988.56 | 30737710.04±17721465.37 |
| Androsterone | C00523 | [M+H]+ | 44820046.83±22114869.37 | 137589880.5±14110430.18 | 116180902.46±37035717.8 |
| Hydroquinone | C00530 | [M]+ | 3763149265.54±1372395152.89 | 4923575591.48±1712011784.12 | 179638868.03±46501378.67 |
| Phenyl acetate | C00548 | [M-H]- | 653051405.24±126732735.07 | 1140576728.46±278893312.94 | 209534356.8±49216806.52 |
| N-Acetylglutamic acid | C00624 | [M+H]+ | 519763.59±62888.69 | 2559052.31±1514991.23 | 15294391.83±4774212.75 |
| Gentisic acid | C00628 | [M+H]+ | 1057225750.92±109660451.18 | 1440551199.48±203304249.33 | 1642247630.72±691722879.77 |
| Creatinine | C00791 | [M]- | 3224232.52±533587.22 | 15831824.28±8906310.18 | 38647581.54±26205805.8 |
| Gulonic acid | C00800 | [M-H2O-H]- | 1323359.67±806748 | 1688097.29±1500697.96 | 2491690.9±2081206.86 |
| 4-Hydroxycinnamic acid | C00811 | [M+H-H2O]+ | 20867158.03±1684641.54 | 59948463.38±5751806.52 | 91781416.65±28632408.39 |
| Tartaric acid | C00898 | [M-H]- | 463923.6±116397.73 | 83179.15±13449.67 | 40992061.11±23499002.77 |
| Dihydrothymine | C00906 | [M]+ | 4197304.45±214050.74 | 37681568.39±36542950 | 63207980.2±52653996.31 |
| 3-Methyladenine | C00913 | [M+H]+ | 49635620.43±24227265.55 | 41805797.34±30026471.07 | 150622357.44±28263795.05 |
| Aminoadipic acid | C00956 | [M+H]+ | 212671497.87±38501335.75 | 90052320.57±11926577.03 | 43593186.86±4027898.39 |
| N-Acetylserotonin | C00978 | [M+H]+ | 95914.42±15890.74 | 328177.36±123981 | 23961682.31±4951775.57 |
| (R)-Pantolactone | C01012 | [M+H-H2O]+ | 7315572.1±2341605.73 | 8995984.36±7467771.51 | 368210075.26±133590753.44 |
| *cis*-4-Hydroxy-L-proline | C01015 | [M+H]+ | 46939036.68±41078607.22 | 19882238.47±11865083.12 | 21060227.76±12632356.49 |
| Dimethylglycine | C01026 | [M-H]- | 86343023.54±10177110.66 | 26622454.11±1785504.06 | 41208363.28±21912296.08 |
| N-Formyl-L-glutamic acid | C01045 | [M]+ | 5514819.67±927676.17 | 179383.37±25009.77 | 5851260.95±6916547.51 |
| 8-Amino-7-oxononanoate | C01092 | [M+H-H2O]+ | 866025.32±626899.6 | 547858.71±33097.92 | 7886781.76±671713.75 |
| 1,2,3-Trihydroxybenzene | C01108 | [M-H]- | 44550667.67±5570852.08 | 15274118.29±389229.61 | 1248371388.58±402019259.59 |
| 3-(2-Hydroxyphenyl)propanoic acid | C01198 | [M-H2O-H]- | 642957380.45±30396766.19 | 42483225.51±2999482.25 | 54735049.53±45179232.74 |
| Dehydroepiandrosterone | C01227 | [M-H2O-H]- | 2797963.8±275568.21 | 3215314.06±320889.83 | 10983174.6±523524.46 |
| Maleic acid | C01384 | [M+H-H2O]+ | 713608209.33±73307946.45 | 677001818.44±277553706.26 | 108894148.46±87977282.36 |
| Gallic acid | C01424 | [M+H]+ | 600036.37±15092.33 | 1034617.06±39105.16 | 14564938.42±9076751.67 |
| Pelargonic acid | C01601 | [M+H]+ | 120266782.34±45723519.69 | 341252659.66±43819703.21 | 20316990.95±520294.72 |
| Piperidine | C01746 | [M+H]+ | 798038133.84±117972692.5 | 2215843527.79±310222482.59 | 71173688.82±49419182.11 |
| Dethiobiotin | C01909 | [M-H]- | 107111.93±20144.14 | 591972.64±49667.25 | 27146297.97±14534714.88 |
| Mandelic acid | C01984 | [M-H]- | 472732.51±493200.46 | 353879.34±19657.11 | 5321922.29±461214.03 |
| L-Erythrulose | C02045 | [M-H2O-H]- | 18164650.82±4232818.42 | 17064518.25±10036256.45 | 10771861.13±1098556.2 |
| Glycylleucine | C02155 | [M+H]+ | 826848.48±309493.81 | 11190578.26±6055182.05 | 63061191.07±41965040.33 |
| 7-Methyladenine | C02241 | [M-H]- | 181432.48±156162.07 | 872796.92±34004.59 | 8382978.82±878746.53 |
| 2-Phenylacetamide | C02505 | [M+H]+ | 1075421.09±14238.13 | 1346570.76±20424.02 | 94786165.85±9459757.02 |
| Pyrimidodiazepine | C02587 | [M]+ | 1842158.21±1101408.95 | 14036974.01±2762877.31 | 159731892.88±51258553.24 |
| Dodecanedioic acid | C02678 | [M-H]- | 660009.75±110508.19 | 1289546.09±842374.45 | 6947007.46±387417.27 |
| Dodecanoic acid | C02679 | [M]+ | 78518471.93±3022550.4 | 95200587.25±18212509.38 | 15474794.81±9423604.33 |
| N-Acetylleucine | C02710 | [M-H]- | 61282.03±13196.06 | 1230915.24±168723.25 | 9275444.86±3053635.19 |
| Hydroxykynurenine | C02794 | [M+H-H2O]+ | 5432896.36±389084.1 | 19690048.89±2742525.12 | 20491160.2±2734880.88 |
| L-Methionine S-oxide | C02989 | [M+H]+ | 19928204.93±5496219.04 | 23248658.75±2113122.17 | 184034104.85±48111300.19 |
| Guanidinosuccinic acid | C03139 | [M]- | 1034053.8±623161.95 | 693766.58±64981.17 | 5255018.54±3135968.63 |
| N5-Methyl-L-glutamine | C03153 | [M+H]+ | 60257498.83±9650414.67 | 85335719.98±5204162.21 | 57857851.83±15172008.25 |
| L-2-Hydroxyglutaric acid | C03196 | [M-H]- | 7218389.04±1672985.45 | 6278027.1±1258502.42 | 33685437.64±17733080.06 |
| L-2,4-diaminobutyric acid | C03283 | [M]+ | 20025817.73±588489.38 | 32365454.1±2120410.74 | 37605853.93±1184733.34 |
| 3-Hydroxybenzyl alcohol glucoside | C03351 | [M]+ | 8328151.72±5316406.72 | 152365138.09±193327593.36 | 4847172.75±4619170.42 |
| 1-Kestose | C03661 | [M-H]- | 4589464.97±1536527.08 | 118593192.17±29899341.12 | 3119024.95±1733016.28 |
| Quinolinic acid | C03722 | [M]+ | 174733598.75±222850258.61 | 71711639.49±67490045.68 | 196877675.45±116467599.62 |
| 3-Methyl-3-hydroxypentanedioate | C03761 | [M+H-H2O]+ | 81420114.26±43633554.45 | 190319510.77±256165758.59 | 297610949.94±151174296.71 |
| (S)-2-amino-6-oxohexanoate | C04076 | [M+H-H2O]+ | 3696547.7±1030031.3 | 14000342.71±11803804.29 | 49012921.44±28086248.37 |
| 13-L-Hydroperoxylinoleic acid | C04717 | [M+H-H2O]+ | 6920392.88±5652329.03 | 46312610.46±6226684.51 | 34301447.53±33862867.44 |
| 13(S)-HpOTrE | C04785 | [M+H]+ | 34831971.24±7807401.43 | 142351312.81±18862126.28 | 16530103±3202761.91 |
| β-Alanyl-L-lysine | C05341 | [M]+ | 11292408.4±11271932.1 | 165980283.16±26683087.61 | 22453563.01±5262991.17 |
| Biocytin | C05552 | [M+H]+ | 82030537.66±2243468.03 | 222221789.08±41510469.98 | 108160761.7±9018152.21 |
| Vanillylmandelic acid | C05584 | [M+H]+ | 31806561.52±13382745.7 | 2311322.36±191200.26 | 12475697.33±271148.97 |
| 2-Phenylethanol | C05853 | [M+H-H2O]+ | 33103461.21±34912019.69 | 113774956.26±27435067.78 | 19885938.95±14176477.72 |
| Pyrrole-2-carboxylic acid | C05942 | [M-H]- | 3270695.74±2338886.18 | 1800969.16±1241351.82 | 542170725.7±135549663.27 |
| Prostaglandin G2 | C05956 | [M+H-H2O]+ | 848420.34±570163.65 | 836923.63±113888.76 | 18664777.44±2622016.51 |
| (S)-Methylmalonic acid semialdehyde | C06002 | [M]+ | 302218122.66±257193991.57 | 153372571.5±147729027.8 | 478132900.85±290291355.67 |
| Terephthalic acid | C06337 | [M-H]- | 6070896.35±6066801.42 | 237004.45±66839.82 | 89868975.82±15445129.58 |
| (9Z,12Z,15Z)-Octadecatrienoic acid | C06427 | [M+H]+ | 1308474.09±515966.3 | 13699842.44±4093521.22 | 22876020.97±3722264.07 |
| 4-Acetamido-2-aminobutanoic acid | C06442 | [M]+ | 600536.45±34430.22 | 6855235.42±531651.75 | 38944494.06±27074723.05 |
| Fluconazole | C07002 | [M+H]+ | 131806.21±98119.73 | 66920.33±5382.65 | 29824842.42±13336764.17 |
| Phenylacetic acid | C07086 | [M+H]+ | 112575776.23±89967378.4 | 50431426.1±17052887.23 | 27878670.77±3795087.47 |
| Orciprenaline | C07144 | [M+H-H2O]+ | 233580.46±30659.23 | 384467.15±34700.27 | 13122899.4±4885954.07 |
| Fomepizole | C07837 | [M+2ACN+2H]2+ | 12861656.72±1321312.08 | 11671626.48±341758.51 | 773596999.72±396908259.43 |
| Metoclopramide | C07868 | [M+H]+ | 20917139.93±1195978.57 | 23817048.75±167071.56 | 10129297.01±7084361.3 |
| Azelaic acid | C08261 | [M-H]- | 2118301.88±1856066.36 | 22182613.49±1441489.45 | 28403826.25±5317843.32 |
| Isovaleric acid | C08262 | [M]+ | 604688738.71±1047351208.04 | 196248013.73±56699527.04 | 602499386.82±209605772.75 |
| Sebacic acid | C08277 | [M-H]- | 95902.3±26272.05 | 1558801.23±1201328.92 | 34598058.32±4153466.18 |
| 2-Heptanone | C08380 | [M]+ | 112907081.98±69805572.83 | 115589491.57±24651625.76 | 42009783.15±14752170.91 |
| Sinigrin | C08427 | [M-H]- | 397153034.81±214240060.03 | 31421702.1±24528985.84 | 36183728.92±29199578.15 |
| Confertifolin | C09376 | [M+H]+ | 16968249.52±2603526.55 | 71554684.09±10146676.22 | 51155542.73±13292042.56 |
| Juvenile hormone III | C09694 | [M+H]+ | 6210754.36±1550142.88 | 29094640.9±1853963.28 | 9205500.14±444130.38 |
| Diphenylamine | C11016 | [M+H]+ | 349002132.53±18428980.18 | 351367985.65±9919569.33 | 16096122.48±1009587.71 |
| Methyl tert-butyl ether | C11344 | [M]+ | 7553105.46±5921331.88 | 39369948.93±41575455.08 | 11483266.83±10268996.2 |
| Methyl jasmonate | C11512 | [M+H]+ | 13339267.08±2094293.35 | 19675419.43±4333974.68 | 92784557.65±38678448.13 |
| (-)-Epigallocatechin | C12136 | [M]- | 40432.46±11670.28 | 2157585.16±37310.4 | 11619690.03±7168601.39 |
| N-α-acetyllysine | C12989 | [M+H]+ | 2005587.67±1663999.14 | 15459473.14±1481996.33 | 177041619.67±43760169.64 |
| 2-Furancarboxaldehyde | C14279 | [M+H]+ | 426658600.64±67523093.13 | 165036207.18±21365447.11 | 239254173.57±29568967.56 |
| 13S-hydroxyoctadecadienoic acid | C14762 | [M+H-H2O]+ | 763619.48±191074.47 | 871951.91±32879.87 | 2211479596.55±1919192100.32 |
| 8,9-DiHETrE | C14773 | [M+H-H2O]+ | 169942.62±96287.14 | 20407691.15±5981369.56 | 24815996.59±6524132.41 |
| 2,3-Dinor-8-iso prostaglandin F2α | C14794 | [M+H-H2O]+ | 50095733.38±23803544.98 | 175150357.72±21144227.04 | 83641437.26±12600490.87 |
| 9(S)-HPODE | C14827 | [M-H]- | 4170160.96±1201503.64 | 2123807.65±1891974.77 | 24695176.55±7056970.97 |
| 9(S)-HPOT | C16321 | [M+H-H2O]+ | 241198913.87±47127052.39 | 967493783.5±131778021.21 | 388165246.4±35545128.19 |
| (10S)-Juvenile hormone III diol | C16505 | [M+H-H2O]+ | 236997.98±26101.47 | 8442634.7±512717.4 | 11213303.31±2212277.55 |
| Citrinin | C16765 | [M+H-H2O]+ | 345018.93±153375.04 | 2184994.95±528534.02 | 10893125.05±4954185.12 |
| Heptanoic acid | C17714 | [M+H]+ | 130619164.24±64027244.85 | 79293227.71±41658348.72 | 54656513.29±4708451.79 |
| (R)-5,6-Dihydrothymine | C21028 | [M]+ | 3855986.3±224498.52 | 16345800.4±21948562.44 | 60887632.1±52183280.75 |
| L-Lactic acid | C00186 | [M+H]+ | 106465443.14±35845539.5 | 180600918.65±20630595.6 | 248789891.16±49757978.23 |
| D-Glucose | C00031 | [M-H]- | 7180631.69±4530274.47 | 1952710.1±1644069.96 | 1913128.04±344894.06 |
| D-Fructose | C00095 | [M-H]- | 8115849.23±4311363.09 | 1047142.24±894781.15 | 889070.91±90511.23 |
| Sucrose | C00089 | [M+H]+ | 674623.75±406062.04 | 1327834.99±1020876.55 | 1840378.7±368075.65 |
| D-Galactose | C00124 | [M-H]- | 15696940.11±4588736.41 | 740099.37±95351.17 | 392072.89±62038 |
